# Deep Eutectic Solvent Assisted Mechano-Enzymatic Preparation for Reprocessable Hot-Melting Starch: A Comprehensive Analysis of Molecular Structure and Thermal Properties

**DOI:** 10.3390/polym17101296

**Published:** 2025-05-09

**Authors:** Xuan Liu, Jia Man, Yanhui Li, Liming Wang, Maocheng Ji, Sixian Peng, Junru Li, Shen Wang, Fangyi Li, Chuanwei Zhang

**Affiliations:** 1 College of Mechanical and Electrical Engineering, Qingdao University, Qingdao 266071, China; 2 Key Laboratory of High Efficiency and Clean Mechanical Manufacture (M of E), School of Mechanical Engineering, Shandong University, Jinan 250061, China; 3 National College of Excellence Engineers, Shandong University, Jinan 250061, China

**Keywords:** hot-melting starch, ball milling, enzymatic hydrolysis

## Abstract

Unlike the hot-melting processing of thermoplastic plastics, the processing of starch-based material relies on the addition of solvents, resulting in their low productivity, hindering large-scale industrialized production. A strategy to realize the high production efficiency of starch-based material, an environmentally friendly modification process without waste liquid generation, was designed to prepare a hot-melting starch (HMS) that can be repeatedly hot melted. Ball milling, enzymatic digestion, and deep eutectic solvent (DES) plasticization modification were combined to prepare the HMS. Ball milling destroyed the starch’s particles and the crystallinity, exposing the hydroxyl group, which allowed amylase to achieve enzymatic hydrolysis more easily. After enzymatic hydrolysis, the molecular chains of modified starch were shortened and the entanglement of molecular chains was reduced, which promoted the slip of molecular chains. The plasticization of DES, which promoted by the broken starch particles and the destroyed crystal structure, formed stronger hydrogen bonds and facilitated hot melting. Furthermore, due to the excellent hot-melting properties, HMS can be combined with sisal fiber and polycaprolactone (PCL) under solvent-free conditions. The tensile strength of HMS/sisal fiber/PCL was increased by 109%; meanwhile, the water contact angle was stabilized at 104°, when the blending ratio of hot-melting starch was 67.5% compared with HMS.

## 1. Introduction

As a product of plant photosynthesis, starch is one of the most commonly used natural bio-based materials. Due to its abundant production, low price, excellent biocompatibility, complete degradation, and polyhydroxy structure, starch has been widely studied in medical supplies, flexible electronics, packaging supplies, and bio-based chemicals [1]. Nevertheless, the entanglement of the molecular chains and the limitation of the crystalline region of natural starch make starch-based materials dependent on a large number of solvents, heat, and mechanical forces in the actual processing and molding process [2,3,4], such as for thermoplastic starch (TPS).

Traditional thermoplastic starch (TPS) is typically prepared using water, glycerol, or other plasticizers under conditions of high temperature and continuous stirring. The conventional plasticizing process consumes significant amounts of water, thermal energy, and mechanical power. In recent years, the extrusion-based preparation of TPS has garnered considerable attention. This method leverages high temperatures, shear forces, and pressure to facilitate the bursting and swelling of starch particles as well as the penetration of plasticizers, thereby completing the preparation of TPS [5]. When TPS is processed, it cannot be reused by reheating and molding, and the processing tolerance is low. Compared with the traditional thermoplastic petroleum-based materials that can be repeatedly molded and are efficient with hot-melting processing technology, the above characteristics of TPS-based composites reduce the production efficiency, and hinder its large-scale industrial application. Hence, developing a hot-melting starch that can repeatedly be molded under the environmentally friendly conditions of a low energy consumption and no waste liquid emission is of great use for the industrial application of starch-based composites.

Modified starches have been widely studied to obtain starch-based materials with better and more stable properties. For example, esterified starch [6], acetylated starch [7], and carboxymethyl starch [8] were obtained by chemical modification. Chemical modification specifically changes the stability, viscosity, solubility, transparency, and other properties of starch, significantly improving the range of starch applications. Yet the chemical modification process produces wastewater containing residual chemical reagents, which harms the environment. In addition, to obtain remarkable properties, the chemical modification process usually needs to achieve a higher degree of substitution, which is demanding on the process. Accordingly, more and more researchers have begun the study of physical and enzymatic modification in recent years. Physical modifications are divided into two categories: heat-treated and non-heat-treated. Typical physical modifications include moist heat treatment, annealing treatment [9], ball milling [10], and ultrasonication [11]. Neither of the physical modification processes altered the hydroxyl functional groups on the starch chains. Ball-milling modification destroys a part of the glycosidic bond and double helix structure of starch, decreasing crystallinity, which can promote the subsequent modification process [10,12]. Enzymatic hydrolysis is widely used to prepare starch derivatives due to its high efficiency and environmental protection. During enzymatic hydrolysis, different amylases specifically hydrolyze different glycosidic bonds. For example, α-amylase specifically hydrolyzes the α-1,4-glucosidic bond, leading to a breakage of the starch chain and an overall decrease in molecular weight [13,14].

In our previous study, it was demonstrated that the entanglement and crystallization zones of complex molecular chains in starch are responsible for limiting the occurrence of hot melting in starch [3]. Consequently, ball-milling modification initially disrupts the crystalline structure of the starch and eliminates part of the molecular chain entanglement within it. The internal structure of ball-milling starch is exposed, enhancing the accessibility of amylase. After the physical modification of ball milling and enzymatic hydrolysis, the crystallinity of starch declines. Meanwhile, as enzymatic hydrolysis modification can reduce the entanglement between molecular chains by reducing the length of starch chains, which is beneficial to promote starch hot melting [15]. Ball milling and enzymatic modification entail no heat loss or solvent waste, being an environmentally friendly modification method and facilitating mass production.

Deep eutectic solvents (DES) are multi-component mixtures of hydrogen bond donors (e.g., amines, amides, carboxylic acids, polyols, etc.) and hydrogen bond acceptors (e.g., quaternary ammonium salts), in which the melting temperatures of the mixtures are lower than those of each component [16]. With the characteristics of thermal stability, non-toxicity, and high solubility, DES has been shown to be a more stable and effective plasticizer than glycerol [17,18,19].

Blending starch with biodegradable hot-melting polymers effectively improves the properties of starch-based composites, such as water resistance and mechanical properties. For instance, Hassan et al. [20] showed that when the content of polylactic acid (PLA) was increased from 0% to 9.72%, the tensile strength of starch/fiber composites was increased from 2.50 MPa to 3.27 MPa. Zhai et al. [21] explored the properties of starch/PBAT composite film and showed that with the increase of poly (butylene adipate-co-terephthalate) content, the tensile strength and elongation at break could reach a maximum of 7.14 MPa and 614%, and the hydrophobic properties were also significantly improved. The polycaprolactone (PCL) is a biocompatible aliphatic polyester with a semi-crystalline state at room temperature. The excellent thermoplasticity, water resistance, and degradability of PCL make it an excellent prospect for new packaging and biomedical applications. Adding plant fibers is usually used to enhance TPS’s mechanical and waterproofing properties. Peng et al. [22] investigated the effect of different lengths of fibers on the properties of TPS and showed that the co-mingled length of 10 mm fibers makes the tensile strength of TPS reach 3.25 MPa, and the co-mingled length of 0.5 mm fibers makes the water contact angle on the surface of the composites reach 130°. The addition of xanthan gum and metal oxides can improve the thermal stability of TPS by increasing its crystallinity [23,24]. Co-mingled chitosan can increase the antimicrobial properties of TPS, which is favorable for the application of starch in packaging materials [25,26]. However, the process of blending starch and other polymer compounds is often dispersed by extruders or solvents, which wastes a lot of energy and time.

In this study, a hot-melting starch (HMS), which can be repeatedly hot melted, was obtained by ball milling, enzymatic hydrolysis under room temperature and low moisture conditions, and DES plasticization, and its good application prospects were demonstrated by compounding it with the degradable hot-melting polymer PCL and sisal fiber. The crystalline structure of starch was destroyed by ball-milling pretreatment, and the winding of the starch chain was reduced by enzymatic hydrolysis modification. This modification method aims to reduce the crystallinity of starch and reduce the restriction of the complex molecular chain entanglement of starch by using the environmentally friendly method. Due to the two-step modification of ball milling and enzymatic hydrolysis, the swelling and rupture of starch particles and the exposure of the internal structure of starch contribute to the plasticization of starch by DES in a simple grinding manner. DES forms a stronger hydrogen bond with enzymatically hydrolyzed ball-milling starch (EBS). Through macroscopic and microscopic characterization, the hot-melting properties and microscopic changes of materials in different modified stages were discussed. The thermal and mechanical properties of different modified samples were compared.

Hot-melting starch can be tightly bonded with other polymer materials by heating without additional processing. Hot-melting starch preparation mixture has the advantages of a high molding efficiency, which is more conducive to the industrial application of starch-based products. The HMS/sisal fiber/PCL composites were simply prepared by using a high-speed powder and hot press, and the water repellency and mechanical properties of the HMS/sisal fiber/PCL composites were tested. A comprehensive study on the modification process and application mode of hot-melting starch was carried out.

## 2. Materials and Methods

### 2.1. Materials

Potato starch was purchased from Macklin Company (Shanghai, China). Anhydrous ethanol (analytical grade) was purchased from Sinopharm Chemical Reagent Co., Ltd. (Shanghai, China), and α-amylase (4 U/mg, the optimum temperature is 45 °C), glycerol, and choline chloride (analytical grade) were purchased from Macklin Company (Shanghai, China). Sisal fiber (The length is about 2 mm) was purchased from Linyi Yuanma Co., Ltd. (Linyi, China). PCL (of a molecular weight of about 5 × 10^4^ Da) was purchased from Shun Jie Plastic Technology Co., Ltd. (Dongguan, China).

### 2.2. The Preparation of Deep Eutectic Solvent (DES)

The preparation of DES was as follows: A mixture of choline chloride and glycerol (molar ratio 1:2) was placed in a sealed glass bottle and stirred by magnetic force at 90 °C until a homogeneous and transparent solution was obtained. Then, the solution was put in a vacuum oven, drying under 100 °C for 1 h to remove moisture. Eventually, DES was obtained.

### 2.3. The Preparation of Hot-Melting Starch (HMS)

Firstly, potato starch was pretreated by ball milling using a planetary ball mill (QM-QX4L, MTIR, Changsha, China). Natural starch (NS) was blended with anhydrous ethanol (1 g starch corresponds to 1 mL anhydrous ethanol) in ball-milling jars, ball milling at 250 rpm for 40 min. Subsequently, the anhydrous ethanol is evaporated to a constant weight at room temperature to obtain ball-milling starch (BS). Distilled water, α-amylase, and BS were blended in a sealed bag at a weight ratio of 1:0.001:1 and stirred evenly. Enzymatic hydrolysis was carried out at room temperature for 1 h to obtain enzymatically hydrolyzed ball-milling starch (EBS). Then, EBS was co-mixed with DES (the mass ratio is 5:1) and ground in a mortar for 10 min to obtain a slurry of HMS. The slurry was dried in an oven at 80 °C for 6 h, and then the HMS in a powder state was acquired by crushing.

Different modified starches were prepared by the above steps: natural starch (NS), ball-milling starch (BS), enzymatically hydrolyzed ball-milling starch (EBS), and hot-melting starch (HMS). In addition, to compare the effects of traditional plasticizers glycerol (G) and DES. The glycerol-plasticized hot-melting starch (EBS-G) was prepared using the above method (the DES in the preparation was replaced with glycerol).

### 2.4. The Preparation of Hot-Melting Starch/Sisal Fiber/PCL Composites

The HMS powder, sisal fiber, and PCL powder were fully co-mingled in different ratios by a high-speed mixer. The HMS/sisal fiber/PCL composites were obtained by hot pressing the blended powders at 0.6 MPa and 100 °C for 5 min, followed by cold molding at 0.6 MPa and 30 °C for 2 min.

### 2.5. Characterization

A scanning electron microscope (SEM; JEOL-JSM6400, Tokyo, Japan) was used to observe the internal microscopic characteristics of different hot-pressed samples.

A polarized light micrograph (PLM; Leica DM4P, Leica, Wetzlar, Germany) was used to characterize the crystal morphology of starch. A total of 0.1 g of starch (dry base) was dissolved in pure water to prepare a 1.0% sample. The sample was loaded onto the sample stage and the magnification of the microscope was adjusted to 100 times.

Size-exclusion chromatography (SEC) was performed using a high-performance liquid chromatograph (Agilent1260, Agilent, Santa Clara, CA, USA). The chromatographic column (Agilent PL Polaegel, 8 μm) was used. In the DMSO mobile phase, the flow rate was 0.8 mL/min, and the temperature was controlled at 35 °C. The molecular weight distribution was tested, and the specific molecular weight was calculated.

Fourier-transform infrared spectroscopy (FTIR; Nicolet iS20, Thermo Fisher Scientific, Waltham, MA, USA) was carried out to investigate the microstructure.

X-ray diffraction (XRD; Miniflex 600, Rigaku, Tokyo, Japan) was used at a speed of 2°/min to characterize the crystalline zone.

The 3,5-dinitrosalicylic acid method [27] was used to determine the reducing sugar value and its dextrose equivalent (DE) to characterize the degree of enzymatic hydrolysis. A UV/visible spectrophotometer (TU-1810, PERSEE, Beijing, China) was used to measure the absorbance at 540 nm, and the DE values were calculated as follows:

DE=reducing sugar expressed glucose (g)/dry weight starch (g) × 100%

Differential scanning calorimetry (DSC250, TA, New Castle, DE, USA) was used to test the thermodynamic properties of materials. Each sample was heated from room temperature to 100 °C, cooled to room temperature, and reheated to 200 °C. Heating was performed at 10 °C/min, and cooling was conducted at 20 °C/min. The second heating scan curve was selected for analysis.

The thermal stability of the materials was investigated by a thermogravimetric analyzer (TGA550, TA, New Castle, DE, USA).

The water contact angle was tested using the contact angle meter (Theta Flex, Biolin, Gothenburg, Sweden).

The other characterization methods about the SEM and XRD of starch at different ball-milling times, the FTIR of glycerol, choline chloride, and DES, are described in Supplementary Materials.

### 2.6. Mechanical Property Tests

Use a universal mechanical testing machine (Instron 3367, ITW, Glenview, IL, USA) for tensile tests in accordance with the ASTM D 638 (Type V) [28]. Five samples of each product were tested at room temperature, and the average value was reported. Samples were stored for 24 h in an environment with a temperature of 20 ± 2 °C and relative humidity of 50 ± 5%.

### 2.7. Statistical Analysis

Each test of the FTIR, XRD, DE value, DSC, TGA, and water contact Angle was performed three times. In the mechanical properties, each sample was tested five times. Differences between samples were compared for statistical analysis using SPSS 23.0 (SPSS Inc., Chicago, IL, USA), one-way analysis of variance (*p* < 0.05).

## 3. Results and Discussions

### 3.1. The Hot-Melting Phenomenon and Preparation Process of Hot-Melting Starch

**Figure 1:**
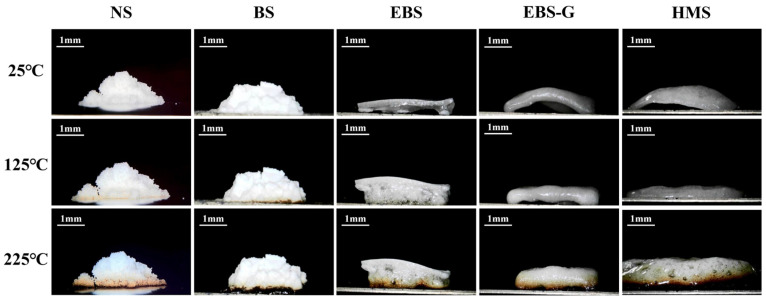
Morphology changes during the heating of NS, BS, EBS, EBS-G, and HMS at 25 °C to 225 °C.

Figure 1 illustrates the temperature-dependent behavioral evolution of the starch samples (NS, BS, EBS, EBS-G, and HMS). Upon thermal treatment, both NS and BS underwent a process of progressive carbonization, without any indication of hot-melting behavior. Significantly, EBS exhibited a unique volumetric expansion specifically at the region of contact with the heating interface. In contrast, the upper non-contact zones of EBS maintained their structural integrity, which clearly demonstrated a spatially differential thermal response within the material’s matrix. As the temperature increased, both the EBS-G and HMS samples showed distinct signs of softening and a subsequent collapse, failing to retain their initial shapes. When compared to EBS-G, the hot-melting behavior of HMS was more pronounced. The hot-melting video of HMS is available in the Supplementary Materials. During the heating process, HMS manifested a clear hot-melting phenomenon, losing its original form and gradually transitioning from a solid state to a flowable one.

**Figure 2:**
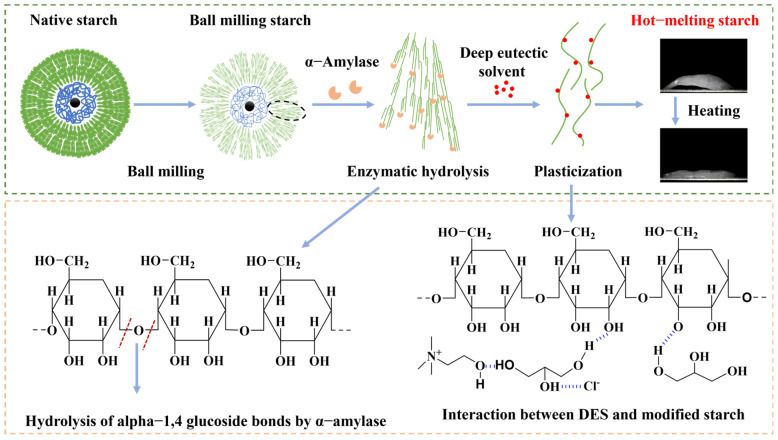
Preparation process of the hot-melting starch.

In order to streamline the enzymatic hydrolysis and plasticization processes involved in the preparation of hot-melting starch, as well as to mitigate environmental pollution and minimize energy consumption during the preparation, this study employed a combined approach of ball-milling modification, room-temperature enzymatic hydrolysis modification, and deep eutectic solvent plasticization. The preparation process of hot-melting starch is illustrated in Figure 2. Firstly, natural starch underwent ball-milling modification as a pretreatment step. This process effectively reduced the starch particle structure and disrupted the crystalline regions [15]. As a result, the ball-milling modification enhanced the susceptibility of starch to α-amylase action under room temperature and low-moisture conditions. Enzymatic hydrolysis serves to shorten the starch chain length, thereby decreasing the entanglement among starch chains. Subsequently, after a simple grinding process, the modified starch was plasticized with DES to obtain the modified hot-melting starch. During the enzymatic hydrolysis modification, starch chains are cut, which reduces the entanglement between molecular chains, thus facilitating molecular chain slippage. Moreover, owing to the incomplete particle structure of EBS, DES can penetrate more readily during the plasticization process, leading to a significant reduction in crystallinity. The molecular entanglement and crystalline domain restrictions inherent in natural starch were effectively disrupted through these three-step modifications to prepare hot-melting starch. Consequently, the material exhibited enhanced flow properties upon heating, ultimately yielding hot-melting starch.

### 3.2. The Morphology of Hot-Melting Starch Products

**Figure 3:**
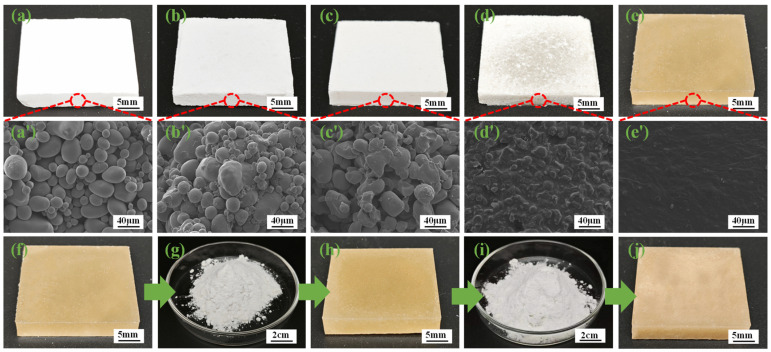
(**a**–**e**’) Macroscopic images and cross-sectional SEM images depicting different modified starches after hot pressing, as follows: (**a**,**a**’) NS, (**b**,**b**’) BS, (**c**,**c**’) EBS, (**d**,**d**’) EBS-G, (**e**,**e**’) HMS, and (**f**–**j**) HMS hot press molding–milled to powder–second hot press molding–milled to powder–third hot press molding.

Macroscopic images and cross-section SEM image depictions of hot-pressed products with different modified samples are shown in Figure 3. The figure shows a remarkable contrasting phenomenon of the hot-melting effect of different modified samples. NS remained intact in the granular state after hot pressing, as shown in Figure 3a,a’. BS with intact starch granules decreased, compared with NS, which was attributed to the destruction of the starch particle structure by ball-milling modification, consistent with the results of the polarizing microscope [15]. As shown in Figure 3c,c’, the boundaries of EBS particles were blurred, and most of the particles were unable to maintain the complete morphology of the starch particles. With the subsequent modification, EBS-G and HMS gradually exhibited smoother cross-sections. After hot pressing, the integrity of the starch granules was gradually reduced, and adhesion was produced at the granule boundaries. The HMS samples present excellent hot-melting results, as shown in Figure 3e,e’, which no longer have an intact particle structure. In addition, the hot-melting effect of glycerol and DES solutions on starch were compared in the figure. It can be seen that the hot-melting effect of HMS (Figure 3e,e’) is better than that of EBS-G (Figure 3d,d’), which was attributed to the better solubility of DES to the starch and the hindering of starch from undergoing recrystallization. Figure 3f–j indicates the repeated hot-melting nature of HMS. After the first and second hot-pressing, the samples displayed a uniform hot-melting effect (Figure 3f,h). The color of the third hot-pressing sample deepened, and the transparency was reduced (Figure 3j), which indicated that part of the starch had undergone thermal decomposition. However, the third hot pressing still obtained the formed samples.

**Figure 4:**
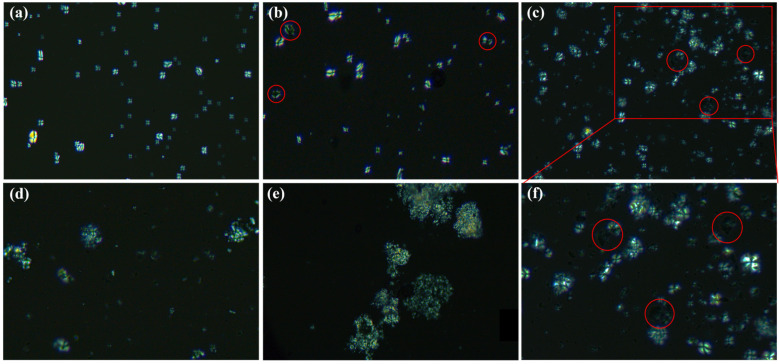
Polarized light microscopy (PLM) images of (**a**) NS, (**b**) BS, (**c**) EBS, (**d**) EBS-G, (**e**) HMS, and (**f**) the magnified region of EBS.

Polarizing microscopy images of starch (NS, BS, EBS, EBS-G, and HMS) are presented in Figure 4. When polarized light traverses through starch particles, the Maltese cross phenomenon is observed in natural starch particles. This phenomenon is a result of the differential scattering and absorption of polarized light between the crystalline and amorphous regions within the starch structure [29]. As illustrated in Figure 4a,b, upon ball milling, the polarization cross effect of BS is noticeably weaker when compared to that of NS. This weakening indicates a change in the crystal structure of the starch. Figure 4c,f presents the polarized light microscopy images of EBS. A portion of the EBS particles exhibit only a faint brightness under polarized light, suggesting that the starch crystal structure has been disrupted [30]. Nevertheless, the majority of the starch particles still display a significant degree of brightness, which can be plausibly attributed to a possible recrystallization process that has taken place. The polarization microscopy images of EBS-G and HMS are shown in Figure 4d,e, respectively. Owing to the presence of the plasticizer, the starch particles become bonded and aggregated. Moreover, EBS-G shows a greater number of crosses, which implies that the structure of HMS has been more thoroughly disrupted in comparison.

### 3.3. Microscopic Performance Analysis

#### 3.3.1. SEC

Table 1 shows the molecular weights of modified starches (NS, BS, EBS, EBS-G, and HMS). The ball-milling treatment disrupts the structural integrity of starch, leading to a reduction in its molecular weight, a decrease in the entanglement of starch chains, and an enhancement of the dispersibility of starch chains. In the process of enzymatic hydrolysis, starch is cut by amylase, the molecular chain is shortened, and the molecular weight is reduced. Due to the higher degree of enzymatic hydrolysis, most of the longer molecular chains are cleaved into relatively shorter molecular chains; therefore, the dispersity of EBS is reduced. In the plasticization process, the hydroxyl groups within the starch form hydrogen bonds with the plasticizer, resulting in a relatively larger molecular structure for the plasticized starch. Consequently, the molecular weights of both EBS-G and HMS are elevated compared to that of EBS. Owing to the higher molecular weight of the DE, the molecular weight of HMS is notably greater than that of EBS-G. These findings collectively demonstrate that ball milling and enzymatic hydrolysis are effective strategies for reducing the molecular weight of starch and alleviating the entanglement between molecular chains. Shorter molecular chains are more prone to peristaltic slippage upon heating. Moreover, the large molecular structure of DES increases the inter-chain spacing of HMS, thereby enhancing its dispersibility, which is conducive to the hot-melting process of HMS.

#### 3.3.2. FTIR

**Figure 5:**
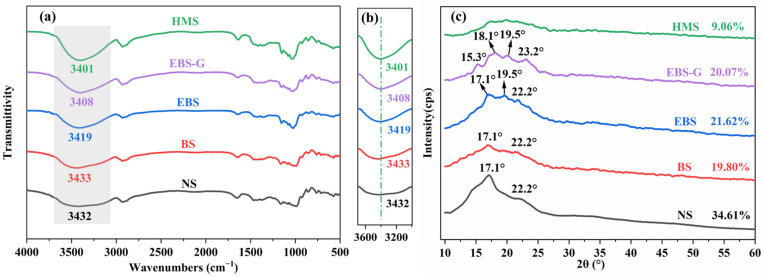
(**a**) FTIR spectra, (**b**) FTIR spectra with localized magnification from 3000 to 3700 cm^−1^, and (**c**) XRD spectra of different samples.

Starch, as a polysaccharide polymer, has a large number of hydrogen bonding groups (H-O⋯H) between its O-H. As shown in Figure 5a, the characteristic peaks in the range of 3570–3050 cm^−1^ are attributed to the O-H stretching vibrations [31]. The hydrogen bonding effect demonstrates that when a hydroxyl group (-OH) participates in new hydrogen bond formation, its stretching vibration wavenumber decreases. According to the simple harmonic oscillator model, the relationship between the force constant and the vibration frequency can be expressed by the formula:

$$\Delta f=f_{\mathrm{nm}}-{\;f}_{m}=\frac{\mu \;(v_{\mathrm{nm}}^{2}-{\;v}_{m}^{2})}{4\pi^{2}}$$

For this, f denotes the force constant, μ represents the reduced mass of the oscillator, v signifies the oscillation frequency, and m/nm indicates the modified/non-modified oscillator. The strength of the hydrogen bond formed is directly proportional to the change in the force constant, which in turn leads to a corresponding alteration in the oscillation frequency. The oscillation frequency, being the product of the wave number and the speed of light, changes due to the generation of new hydrogen bonds, which alters the wave number. Additionally, since the formation of hydrogen bonds is accompanied by a reduction in the force constant, the wave number of the vibrational region in the infrared spectrum shifts towards lower values [32]. Compared to NS and BS, the O-H stretching vibrational peaks of EBS, EBS-G, and HMS all shifted in the low-frequency direction to 3419, 3408, and 3401 cm^−1^, respectively. This indicated that new hydrogen bonds stronger than the original ones are formed during the modification process. In addition, HMS showed the most significant movement, suggesting that the addition of DES led to the formation of more stable hydrogen bonds than glycerol, which helped to inhibit the recrystallization properties of starch. In the wavenumber range of 3050–3570 cm^−1^, EBS exhibited vibration peaks that were both stronger and steeper compared to those of BS. This observation suggested that EBS possesses an enhanced starch chain mobility and more active hydrophilic groups [3]. The enzymatic hydrolysis modification process was responsible for promoting the mobility of starch chains. Additionally, it was noted that the peak intensity and steepness of HMS exceeded those of EBS-G. This indicated that DES, when used as a plasticizer, endows the modified starch with a greater number of mobile starch chains. The increased mobility of starch chains in HMS compared to EBS-G provides a rational explanation for the superior hot-melting fluidity of HMS.

#### 3.3.3. XRD

The change in crystallinity during the modification process was investigated using XRD. The crystallinity of each sample was calculated using Jade 9 software. As shown in Figure 5c, natural potato starch exhibits strong diffraction peaks at 17.1° and 22.2°, which are characteristic of B-type starch [33]. Compared with NS, the diffraction peak intensity of BS here is reduced, and the corresponding crystallinity is also reduced by 14.81% (from 34.61% to 19.80%). This phenomenon indicates that the ball-milling treatment destroys the ordered crystal structure of starch, resulting in a decrease in crystallinity and a gradual transformation into an amorphous structure [34]. Ball-milling pretreatment is helpful for the subsequent modification. A new diffraction peak appears in EBS at 19.5°, indicating that EBS has both B and V type structures. In the process of the enzymatic hydrolysis of ball-milling starch, the endogenous fat of starch precipitates with amylose to form an amylose–lipid structure [35]. At the same time, the crystallinity of EBS increased slightly compared with that of BS, which might be due to the recrystallization process of starch during the removal of water after enzymatic hydrolysis. The decrease in the crystallinity of EBS-G was not significant, only 1.55% (from 21.62% to 20.07%) compared to EBS. According to previous reports, the crystalline type of potato starch may change into type A or B + A after modification [36], which explains the new diffraction peak phase of EBS-G at 15.3° and 23.2°. As the storage time increases, glycerol is separated from EBS-G due to the weaker interaction between glycerol and starch. Therefore, the obstruction of glycerol to the recrystallization of starch is insignificant, and the reduction of crystallinity is not apparent. There are no obvious diffraction peaks on the XRD curve of HMS. The crystallinity of HMS was reduced by 12.56% (from 21.62% to 9.06%) compared to EBS. The above results indicate that the ball-milling treatment not only destroys the particle structure of natural starch, but also causes certain damage to the crystalline structure of natural starch, making it easier for amylase to carry out enzymatic hydrolysis modification at low moisture and room temperature. In the previous report, the DES plasticization of natural starch failed to significantly reduce its crystallinity because of the intact granular structure of natural starch, and DES could only penetrate into the surface region of the starch [37]. When contrasted with glycerol, HMS exhibits a reduced crystallinity. This observation implies that a stronger and more compact hydrogen bond has been established between the DES and the starch molecules. This robust hydrogen bonding significantly disrupts the crystallization regions within the starch structure. As a consequence, the restrictive influence of the crystallization regions on the mobility characteristics of the starch chains is substantially diminished. With the reduced hindrance from the crystalline region, the thermal melting of HMS is effectively facilitated, enabling it to reach its molten state more readily under appropriate thermal conditions.

### 3.4. DE Value

**Figure 6:**
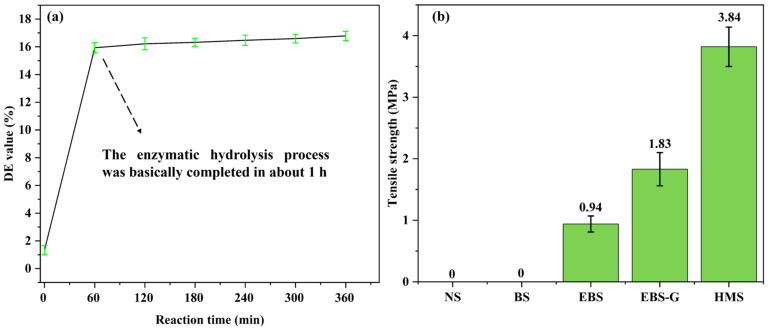
(**a**) DE values of starch at different enzymatic hydrolysis times, and (**b**) the tensile strength of different samples.

The DE value of starch hydrolyzed by enzymes reflects the proportion of reducing ends exposed during enzymatic hydrolysis and is an indicator for evaluating the degree of enzymatic reaction [13]. DE values with the time-lapse of enzymatic digestion are shown in Figure 6a. The DE value increased rapidly during the first hour and grew from an initial 1.3% to 15.9% at 1 h. With time, the DE value of enzyme hydrolysis for 5 h was 16.4%, and the change was not obvious. This phenomenon indicates that under the conditions of enzymatic hydrolysis in this paper, amylase has been basically deactivated in about 1 h. The experiment did not require too long a enzymatic hydrolysis time. Therefore, 1 h was chosen as the time of enzymatic hydrolysis.

### 3.5. Thermal Properties Analysis

#### 3.5.1. DSC

**Figure 7:**
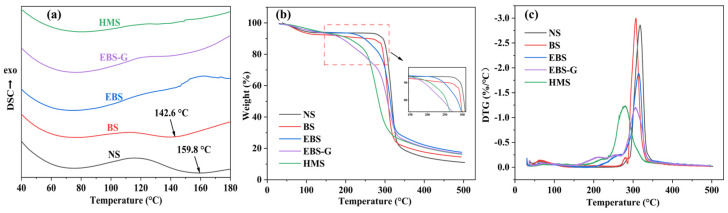
(**a**) The DSC, (**b**) TGA, and (**c**) DTG of different samples.

The test results for NS, BS, EBS, EBS-G, and HMS are presented in Figure 7a. Differential Scanning Calorimetry (DSC) was employed to investigate the thermal energy absorption/release associated with phase transitions and chemical reactions in the materials. The observed endothermic transitions correspond to the melting of the starch microcrystalline structure, where broader endothermic peaks correlate with higher enthalpy changes. This observation suggests that materials exhibiting wider melting endotherms possess more thermally stable crystalline structures that require a greater energy input for disruption during heating [3]. The initial temperature (T_o_), peak temperature (T_p_), termination temperature (T_c_), and enthalpy change ΔH of the first hot-melting peak of different products are shown in Table 2.

Generally, the endothermic transition of 50–80 °C is considered to be the complete gelatinization of starch, and the enthalpy change of starch gelatinization reflects the heat required for the dissociation of the double helix structure of starch [38]. When the peak temperature is around 75 °C, the thermal melting peaks ΔH of NS and BS are measured to be 47.7 J/g and 39.9 J/g, respectively. The magnitude of the heat absorption peak is directly related to the amount of residual microcrystalline structure within the starch samples. Specifically, a larger heat absorption peak indicates a greater presence of the residual microcrystalline structure. The relatively smaller enthalpy change observed in BS suggests that the ball-milling process has indeed disrupted the crystalline structure of natural starch. The ΔH of EBS is 41.5 J/g, slightly higher than that of BS. This is because of the recrystallization of the original hydrogen bond sites of starch due to the moisture present during enzymatic hydrolysis and the subsequent drying. In the XRD test, it is manifested that the crystallinity of EBS is higher than that of BS. Upon undergoing the plasticization process, both EBS-G and HMS exhibited reduced enthalpy changes. This observation suggests that the plasticizer effectively alters the crystal structure of the starch. In particular, the enthalpy change of HMS was found to be a mere 27.2 J/g. This low value implies that the DES, as the plasticizer, inflicts more substantial damage to the crystal structure of HMS. Consequently, a lesser amount of heat is required for the dissociation of HMS, highlighting the significant impact of DES on weakening the intermolecular forces within the starch and facilitating its transition to a more fluid state during the melting process.

NS and BS showed heat absorption peaks near 159.8 °C and 142.6 °C, respectively, in the range of 120–180 °C as shown in the figure, and the heat absorption peaks in this range were related to the hot melting of the crystalline region formed by the amylose [39]. Compared to NS, the peak temperature of BS was shifted towards lower temperatures, which was attributed to the disruption of the amylose glycosidic bonds during the ball-milling process, which induced the breakage of the amylose and disrupted the crystalline zones of the amylose. No significant heat-absorption peaks were observed in the range of 120–180 °C for EBS, EBS-G, and HMS, which was attributed to the fact that α-amylase hydrolyzed a large amount of α-1,4-glucosidic bonds of amylose during the enzymatic hydrolysis process. The hydrolysis of the glucoside bond causes the amylose chain to break, and the crystallization zone is destroyed in large quantities, so the heat absorption in the heating process is less, and no obvious heat absorption peak can be formed.

#### 3.5.2. TGA

Figure 7b,c presents the TGA and DTG curves of various modified starch samples. Table 3 lists five crucial temperature characteristics: the onset temperature at which the sample weight loss becomes observable (T_onset_), the temperature at which the absolute value of the inverse weight curve reaches its maximum (T_max_), and the temperatures corresponding to 5% weight loss (T_d5_) and 30% weight loss (T_d30_) [32,40]. Notably, the T_onset_ values of EBS, EBS-G, and HMS are significantly lower than those of NS and BS. In particular, the T_onset_ of HMS drops to 245.4 °C. This phenomenon can be attributed to the enzymatic hydrolysis process, which shortens the starch chain length, consequently reducing its thermal stability. Given that the DES has a superior solubility for starch and a better resistance to recrystallization compared to glycerol, the thermal stability of HMS is lower than that of EBS-G. However, within the temperature range of 160–200 °C, the EBS-G curve exhibits a transient decline due to the thermal decomposition of glycerol, a feature that is absent in the HMS curve. This disparity can be accounted for by the fact that DES plasticizers containing added choline chloride possess a higher thermal stability than glycerol. The T_max_ values also adhere to the aforementioned pattern. However, the T_max_ of EBS is slightly higher than that of BS, a difference that is associated with the recrystallization process.

### 3.6. Mechanical Properties

The tensile properties of different hot-pressed samples were investigated, and the results are shown in Figure 6b. Because the samples obtained by the direct hot pressing of NS and BS powders under solvent-free conditions are very loose in texture and fragile, they cannot meet the requirements for mechanical property testing. Therefore, the measurements in Figure 6b are shown as 0 MPa. With the progress of modification, the hot-melting performance is improved. The increasing bonding within the sample results in increased mechanical properties. The best mechanical properties of the HMS hot-pressed samples were found to be 3.84 MPa for tensile properties, respectively. According to previous reports, DES (choline chloride–glycerol) has a better plasticizing effect than glycerol, and the large size of DES is conducive to improving the fluidity of starch chains, so the DES plasticized starch shows better mechanical properties [19], which is consistent with the measured results of this study. The outstanding hot-melting properties result in tight internal bonding, leading to better mechanical properties than other samples.

### 3.7. The Properties of Hot-Melting Starch/Sisal Fiber/PCL Composite Samples and Molded Products

The obtained mixture is designed as HMS-x (x is the weight ratio of HMS to PCL, and the weight ratio of HMS and PCL to sisal fiber is fixed to 9:1, and it is important to note that HMS-0 and HMS-100, respectively, denote the compositions of 90% PCL + 10% sisal fiber and 90% HMS + 10% sisal fiber in the composite material). The blending ratios of different samples prepared in this paper are shown in Table 4.

**Figure 8:**
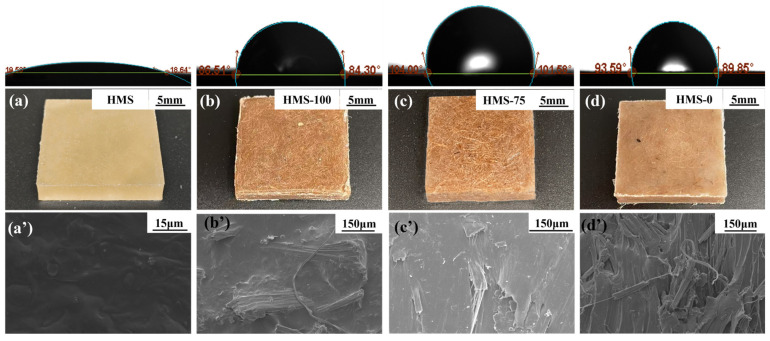
Macroscopic images and cross-sectional SEM images: (**a**,**a**’) HMS, (**b**,**b**’) HMS-100, (**c**,**c**’) HMS-75, and (**d**,**d**’) HMS-0.

**Figure 9:**
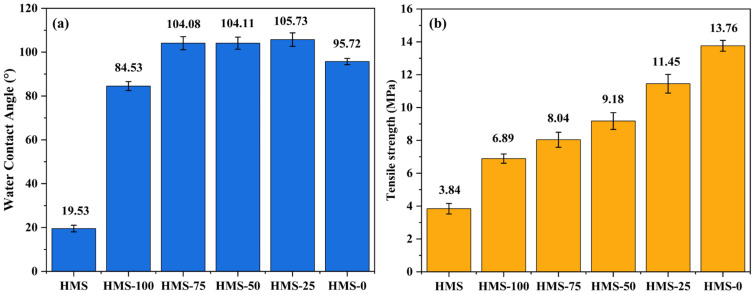
(**a**) The water contact angle and (**b**) tensile strength of different samples.

HMS has the capability to undergo hot-melt lamination with other degradable hot-melting polymers in the absence of solvents. The properties of the HMS/sisal fiber/PCL composites, which are blended at various ratios, are illustrated in Figure 8. As can be discerned from the SEM image (a), due to complete hot melting, HMS exhibits a uniform and smooth cross-section. In the case of HMS-100, the HMS matrix firmly encapsulates the sisal fibers, and there is no noticeable separation between them. The composite sample consisting of PCL and sisal fiber is shown in Figure 8d. Evident gaps are present between the PCL and the sisal fiber, which indicates that PCL is unable to effectively encapsulate the sisal fiber. In sharp contrast, in HMS-100, no significant separation is observed among HMS, sisal fiber, and PCL. This suggests that HMS can be successfully fabricated through hot pressing after a simple stirring and blending process. Furthermore, owing to its hot-melting characteristic and cohesiveness, HMS demonstrates an excellent interfacial compatibility with other polymer materials. At the same time, as depicted in Figure 9, the incorporation of sisal fiber leads to an increase in the tensile and waterproof properties of the HMS materials by 79.4% and 332.8%, respectively. This clearly shows that sisal fiber significantly enhances the properties of the HMS-based materials. The SEM images of HMS-75 and HMS-0 are presented in Figure 8c,c’,d,d’). When compared with HMS-0, the cross-section of HMS-75 has a smaller number of pores. This further confirms that HMS can be used to prepare composites with PCL and sisal fiber via hot-melting blending.

Figure 9a presents the water contact angles of diverse samples. HMS exhibits a poor hydrophobicity, characterized by a low water contact angle of 19.53°, predominantly because of the abundance of hydrophilic -OH groups on its surface. Conversely, the incorporation of sisal fibers instigates a remarkable transformation: the water contact angle of the HMS-100 composite sharply escalates to 84.53°, marking a 332.8% increase relative to pure HMS. For other HMS/sisal fiber/PCL composites, their water contact angles remain stably above 104°, slightly exceeding the 95.72° of the HMS-0 sample. This enhancement can be ascribed to the strengthened interfacial bonding among HMS, sisal fibers, and PCL, which gives rise to a more compact surface structure, a finding that aligns with the observations from SEM analysis.

The physical surface morphology images and SEM images of the composite materials, which are provided in the Supplementary Materials, further elucidate these phenomena. The addition of sisal fibers generates a specific microstructure on the surface of HMS-100. The increased surface roughness contributes to the elevation of the water contact angle [22]. Moreover, the outstanding fluidity of PCL allows a layer of PCL film to coat the surface of the HMS/sisal fiber/PCL composite. This film not only preserves the microstructure imparted by the sisal fibers but also diminishes the contact area between starch and water, thereby further augmenting the water contact angle. In the case of HMS-0, its water contact angle is comparable to that of PCL because the excellent fluidity of PCL prevents the formation of a microstructure by sisal fibers on its surface. Instead, the surface consists solely of a PCL sealing layer. Figure 9b shows the tensile strength of HMS matrix composites, which is significantly enhanced by the addition of sisal fiber and PCL. As the proportion of PCL increases, the tensile properties of the composite are further strengthened. When large amounts of PCL were added, the tensile strength of the composite reached 11.45 MPa, which was attributed to the excellent mechanical properties and fluidity of PCL. The tensile strength of HMS-75 is increased by 109% compared with that of HMS, which proves that the mechanical properties of HMS can be significantly improved by adding PCL and sisal fiber.

With the addition of sisal fiber and PCL, the mechanical properties and water resistance of HMS were considerably improved. When the ratio of HMS was 67.5%, the tensile strength of HMS-75 was 8.04 MPa, which was 109% higher compared with the tensile strength of HMS (3.84 MPa). Moreover, with the further increase of the PCL addition, the tensile strength of HMS-25 reached 11.45 MPa, respectively. The water contact angle of the composites with different ratios is stably greater than 104°.

## 4. Conclusions

A new method was provided for the preparation of hot-melting starch: ball milling, enzymatic hydrolysis at room temperature, and DES plasticization. The combination of ball milling and enzymatic hydrolysis can effectively reduce the entanglement of molecular chains and the restriction of the crystallization zone. On the basis of destroying the crystal structure of starch, DES as plasticizer penetrated into the interior of starch, further destroyed the crystal structure of starch, and improved the ductility of modified starch. The plasticization of DES prevents the recrystallization of starch. HMS has strong hydrogen bonding and a resistance to recrystallization. Due to the reduction of molecular chain entanglement, molecular weight, and the crystallization zone restriction of HMS, sufficient conditions are provided for the peristaltic slip of molecular chains within HMS, so that HMS can achieve hot melting before reaching the thermal decomposition temperature.

Furthermore, HMS enhances its collaborative processing capabilities with other degradable polymers. The composite material produced through hot pressing with sisal fiber and PCL powder under solvent-free conditions exhibits outstanding mechanical properties and hydrophobicity. HMS demonstrates excellent fluidity, effectively enveloping the sisal fiber after hot melting. The flow of hot-melting starch and PCL ensures a tight internal bonding within the composite material, eliminating pores, resulting in a well-integrated composite sample. Superior interfacial compatibility contributes to the composites’ properties.

This study provides a convenient new way of blending starch with other biodegradable hot-melting materials, which improves productivity and processing tolerance and expands the advantages of starch-based material applications. In the continuity of this work, we will further explore the compatibility of HMS with other degradable hot-melting polymers and improve the stability, expanding the application prospects of HMS-based composites.

## Figures and Tables

**Table 1:** The molecular weight of NS, BS, EBS, EBS-G, and HMS.

Materials	Mn (×10^3^ g/mol)	Mw (×10^3^ g/mol)	Dispersity (×10^−2^)
NS	932.3 ± 1.1	5265.4 ± 1.3	564.7 ± 0.1
BS	778.7 ± 1.3	4683.4 ± 1.5	601.4 ± 0.1
EBS	242.0 ± 2.0	1279.4 ± 2.0	528.8 ± 0.1
EBS-G	242.9 ± 0.8	1290.0 ± 1.0	531.2 ± 0.1
HMS	261.2 ± 1.0	1419.5 ± 1.1	543.5 ± 0.1

Mn: number-average molecular weight, Mw: weight-average molecular weight, Dispersity: Mw/Mn.

**Table 2:** Thermal properties of the first hot-melting absorption peaks in different samples.

Materials	T_o_ (°C)	T_p_ (°C)	T_c_ (°C)	ΔH (J/g)
NS	33.6 ± 0.3	75.8 ± 0.3	108.3 ± 1.3	47.7 ± 2.4
BS	33.9 ± 0.1	77.9 ± 0.5	107.4 ± 1.3	39.9 ± 1.4
EBS	34.4 ± 0.2	74.8 ± 0.2	115.8 ± 0.9	41.5 ± 1.4
EBS-G	35.7 ± 0.3	77.0 ± 0.1	116.8 ± 1.5	36.5 ± 1.7
HMS	35.3 ± 0.1	80.4 ± 0.2	110.3 ± 2.4	27.2 ± 1.1

T_o_: initial temperature, T_p_: peak temperature, T_c_: end temperature, ΔH: enthalpy change.

**Table 3:** TGA data of NS, BS, EBS, EBS-G, and HMS.

Materials	T_onset_ (°C)	T_max_ (°C)	T_d5_ (°C)	T_d30_ (°C)	Residue (%)
NS	287.0 ± 0.6	317.6 ± 0.7	300.0 ± 3.3	313.2 ± 1.5	11.0 ± 1.6
BS	281.4 ± 2.6	307.2 ± 2.3	293.5 ± 1.9	305.1 ± 1.8	14.5 ± 3.3
EBS	261.4 ± 4.7	314.1 ± 0.4	280.3 ± 0.5	309.7 ± 1.0	17.5 ± 0.7
EBS-G	259.9 ± 1.7	306.5 ± 1.0	279.4 ± 2.0	309.4 ± 0.4	16.6 ± 2.2
HMS	245.4 ± 1.5	280.1 ± 3.6	257.2 ± 2.3	281.0 ± 1.2	16.3 ± 1.2

T_onset_: the temperature where the loss of the sample weight began to be visible, T_max_: the temperature where the absolute value of the inverse weight curve was at its maximum, T_d5_/T_d30_: the temperatures at 5/30% weight loss.

**Table 4:** The composition of different materials.

Samples	HMS (%)	PCL (%)	Sisal Fibers (%)
HMS-0	0	90	10
HMS-25	22.5	67.5	10
HMS-50	45	45	10
HMS-75	67.5	22.5	10
HMS-100	90	0	10
HMS	100	0	0

## Data Availability

Data are contained within the article and Supplementary Materials.

## Supplementary Materials

The following supporting information can be downloaded at https://www.mdpi.com/article/10.3390/polym17101296/s1, Figure S1. SEM diagram of starch after different milling time: (a) 10 min, (b) 20 min, (c) 30 min, (d) 40 min; Figure S2. XRD pattern of starch after different milling time; Figure S3. Morphology of starch particles after enzymatic hydrolysis; Figure S4. FTIR of choline chloride, glycerin, and DES; Figure S5. SEM images and physical images of the surface structure of (a,a’) HMS-0, (b,b’) HMS-50 and (c,c’) HMS-100; Video S1. Hot-melting video of HMS.

## Abbreviations

The following abbreviations are used in this manuscript:

NS	Natural starch
BS	Ball-milling starch
EBS	Enzymatically hydrolyzed ball-milling starch
DES	Deep eutectic solvent
EBS-G	Glycerol-plasticized hot-melting starch
HMS	Hot-melting starch
PCL	Polycaprolactone
