# Supplementary material for: Deep Eutectic Solvent Assisted Mechano-Enzymatic Preparation for Reprocessable Hot-Melting Starch: A Comprehensive Analysis of Molecular Structure and Thermal Properties
Source: Polymers (Basel). 2025 May 9;17(10):1296. doi: 10.3390/polym17101296 (PMC12114732; doi:10.3390/polym17101296)
Supplement: Supplementary file 1 [file polymers-17-01296-s001.zip › Supplementary material.pdf]

Supplementary Material

Deep eutectic solvent assisted mechano-enzymatic preparation for reprocessable hot-melting starch: a comprehensive analysis of molecular structure and thermal properties

Xuan Liu [a], Jia Man [b], Yanhui Li [a], Liming Wang [b], Maocheng Ji [b], Sixian Peng [b], Junru Li [a, *], Shen Wang [b, *], Fangyi Li [b], Chuanwei Zhang [a, *]

[a.] College of Mechanical and Electrical Engineering, Qingdao University, Qingdao, 266071, China

[b.] Key Laboratory of High Efficiency and Clean Mechanical Manufacture (M of E), School of Mechanical Engineering, Shandong University, Jinan, 250061, China

[c.] National College of Excellence Engineers, Shandong University, Jinan, 250061, China

*Corresponding authors:
Chuanwei Zhang, Email: zhangchuanwei3722@163.com

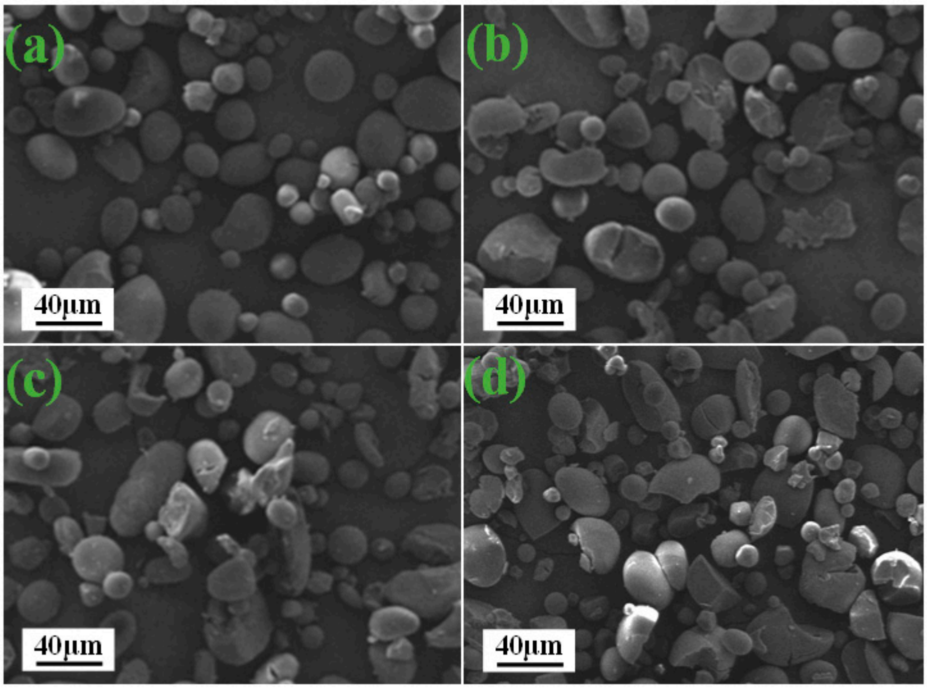

Figure S1. SEM diagram of starch after different milling time: (a)10 min, (b)20 min, (c)30 min, (d)40 min.

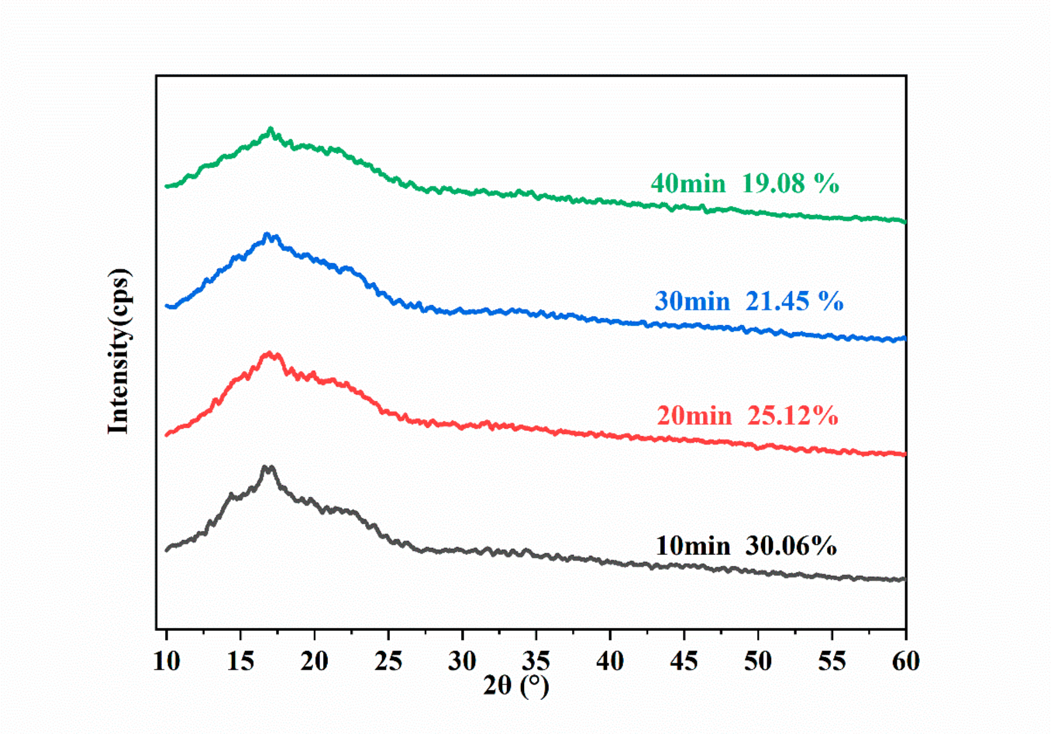

Figure S2. XRD pattern of starch after different milling time.

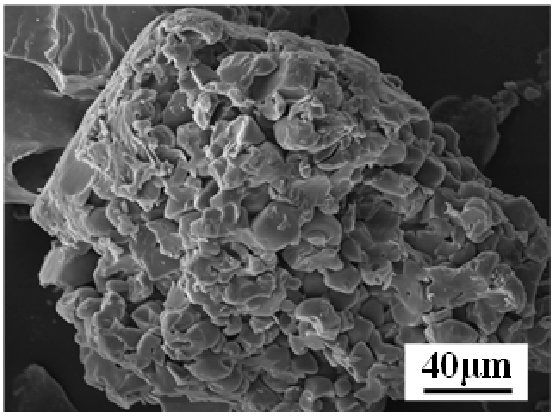

Figure S3. Morphology of starch particles after enzymatic hydrolysis.

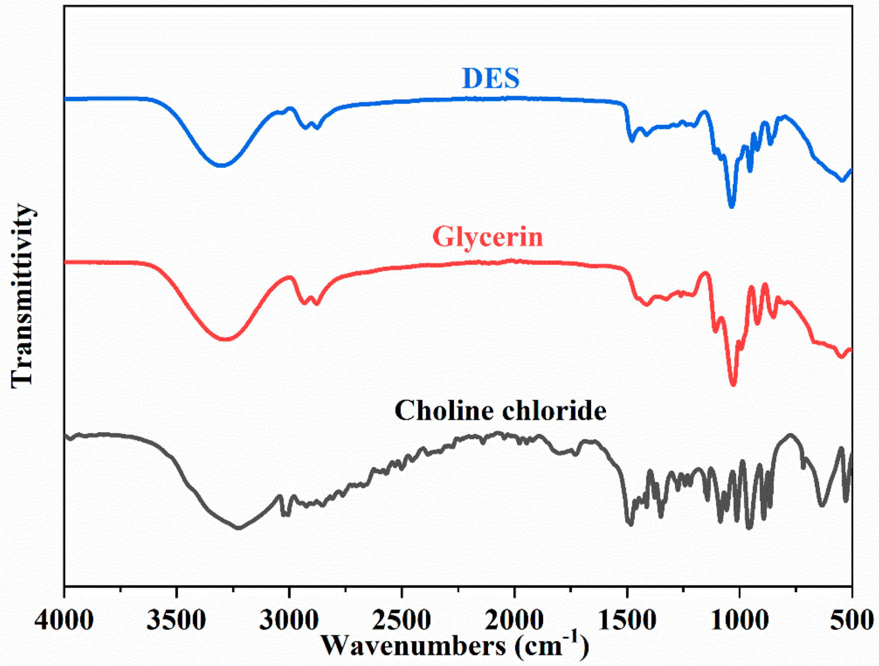

Figure S4. FTIR of choline chloride, glycerin, and DES.

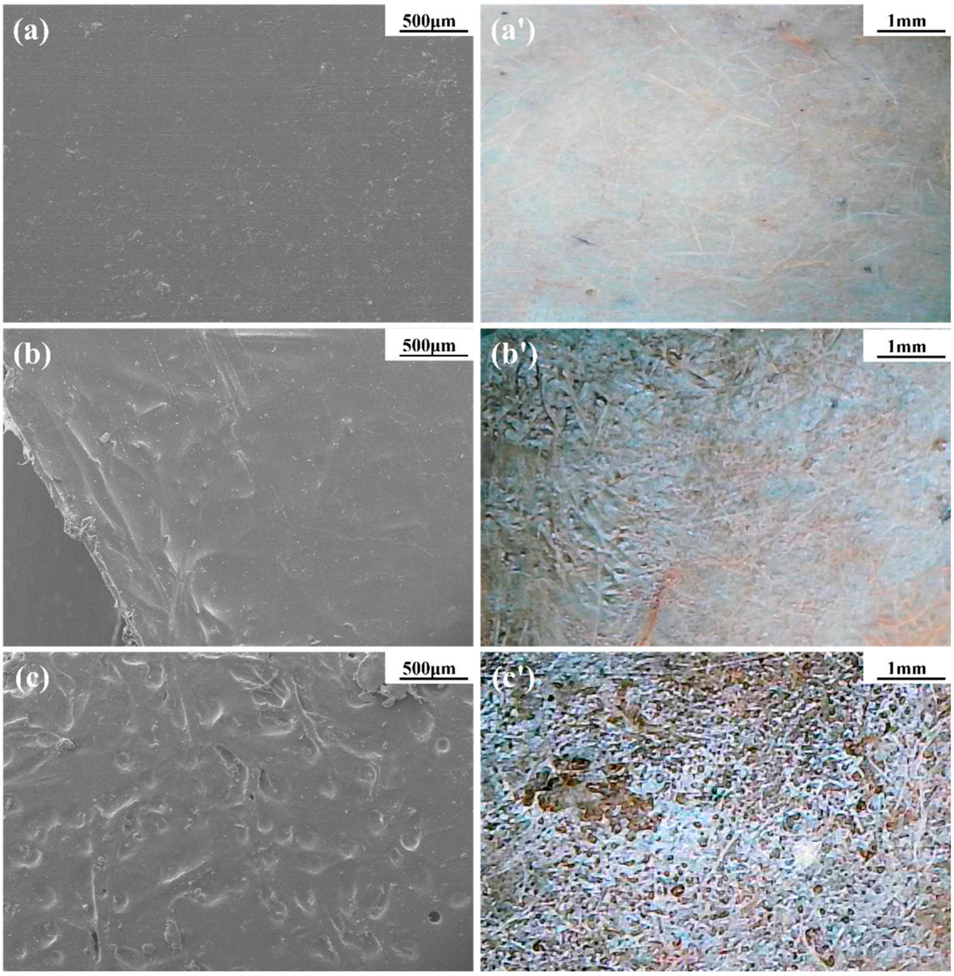

Figure S5. SEM images and physical images of the surface structure of (a, a') HMS-0, (b, b') HMS-50 and (c, c') HMS-100
